# Community involvement in biomedical research conducted in the global health context; what can be done to make it really matter?

**DOI:** 10.1186/s12910-018-0283-4

**Published:** 2018-06-15

**Authors:** Federica Fregonese

**Affiliations:** Centre de Recherche du CHUM, Montréal, Canada

**Keywords:** Community involvement, CAB, Clinical trials

## Abstract

**Background:**

Community involvement in research has been advocated by researchers, communities, regulatory agencies, and funders with the aim of reinforcing subjects’ protection and improving research efficiency. Community involvement also has the potential to improve dissemination, uptake, and implementation of research findings. The fields of community based participatory research conducted with indigenous populations and of participatory action research offer a large base of experience in community involvement in research. Rules on involving the population affected when conducting research have been established in these fields. But what is the role of community engagement in clinical research and observational studies conducted in biomedical research outside of these specific areas?

**Main body of the abstract:**

More than 20 years ago, in the field of HIV medicine, regulatory bodies and funding agencies (such as the US National Institutes of Health) recommended the constitution of a formal organism, the Community Advisory Board (CAB), as part of the study requirements for HIV trials. More recently, CABs have been adopted and used in other fields of medical research, such as malaria. CABs are not without limitations, however, and there is little research on the effectiveness of their use in achieving community protection and participation. Nevertheless, CABs could be a model to import into clinical trials and observational research where no alternative model of community representation is currently being used.

**Conclusions:**

Allocating more resources to training and shifting more power to community representatives could be part of the solution to current CAB limitations. However, for researchers to be able to apply these recommendations on community involvement, certain conditions need to be met. In particular, funding agencies need to recognize the human and financial resources required for serious community involvement, and the academic environment needs to take community involvement into account when appraising, mentoring**,** and training researchers.

## Background

### Preface

After my studies and training in clinical medicine, my first experience in research was with an international research unit conducting a clinical trial on HIV prevention. As researchers, we were asked by the funding agency to institute and meet regularly with a community advisory board (CAB). Putting together and working with this body of community representation was a new challenge for researchers involved in the study. First, close collaboration with the local partners was required to identify community members who could serve on the board. Then, the interaction between the two worlds—scientists from the North and community members from the South, speaking “different languages” in so many ways—was not always easy or immediate. But with the passage of time, the two bodies became more accustomed to each other. Researchers learned to listen to the CAB’s suggestions and comments on their work. They also benefited from the CAB’s help in finalizing tools such as the consent form and in disseminating information about the study, which resulted in more efficient recruitment.

Later, continuing my research work in other fields of global health—quite different from the world of HIV clinical trials—I was confronted with the fact that organized community involvement was not formally required for observational or other interventional biomedical research.

The CAB system, which at times, and for different reasons, may be seen as an imperfect way to create a partnership between researchers and the community, seemed a better solution for informing and giving a voice to the community affected by the research, as compared to there being no formal system at all.

In this paper, inspired by this reflection, my aim is to briefly describe the ethical needs for community involvement in any type of public health research, to explore the gap between those needs and the tools available to researchers to fill them, and to suggest possible solutions to meet these needs.

### Introduction

An extensive literature underscores the need for communities to be involved in research [1, 2], arguing that a “community” dimension should be considered along with the “individual” one in human subject protection in research [2–5]. Building upon the experience of research in aboriginal communities, community based participatory research (CBPR) [2, 6, 7] is “a collaborative research approach that is designed to ensure and establish structures for participation by communities affected by the issue being studied, representatives of organizations, and researchers in all aspects of the research process to improve health and well-being through taking action, including social change” [8]. CBPR advocates equal partnership between researchers and community.

Similarly, in participatory action research (PAR), communities take action to improve their own health and become “owners” of the research process. Through power sharing and active participation of research subjects, PAR focuses on research that enables actions for change [9].

While CBPR and PAR have been adopted in several areas of research, they do not apply to all types of studies, and not all health (including public health) research is conducted as CBPR [5] or PAR. Nevertheless, in this paper, I argue that biomedical research in public health areas, developed on any specific disease, could—and should—involve communities affected by the disease under investigation. This is especially the case in the field of global health, where researchers do not belong to the populations under study.

Possible solutions described here are taken from the example of HIV medicine, where activism has advanced the agenda of community involvement as a way to advocate for relevant research and ensure strong vigilance for the safety of participating subjects.

The target audience for this paper is the community of researchers in biology, medicine, and epidemiology who study a particular disease or health risk, conducting both interventional and observational research—whether longitudinal, as in cohort studies, or cross-sectional, as in repeated surveys.

Relationships between communities and research take different forms, ranging from community consultation in specific stages of the research, to community representation during the whole research process, and even to a long-term and more complex partnership. Different types of community involvement could be appropriate in different situations; for example, informal consultations could be sufficient in some studies, but in others, especially if more vulnerable populations are affected, there could be cause for more formal consultation or partnership [10].

This paper is not a intended to be a comprehensive analysis of all possible forms of community involvement in research, but rather a reflection on why and how community involvement should be considered in fields of biomedical research where participatory research is not routine practice. For the purpose of this paper, the term “community involvement” is used to indicate different forms of community consultation and representation in research.

In summary, in this article, I focus on why community involvement should be a requirement in the fields of biomedical research in public health and global health, what tools are available to researchers for involving communities in studies not conducted in a CBPR or PAR context, the limitations of these currently available tools, and ideas to overcome those limitations.

## Main text

### Part I: Arguments for community involvement in public health research

Community involvement in research can be advocated from different perspectives. There are three main arguments, discussed below, to support the need for community involvement, all of which can apply to different fields of public health research.

First, informing and consulting members of the broader community on the ongoing research is seen as an additional protection for ethical conduct of the research besides that provided by ethics committee approvals and informed consent [3, 5]. Communities can act as gatekeepers [11] for the principles of respect for persons, beneficence, and justice, as affirmed by the Belmont report [12], extending their meaning to the people affected by the research, even if they are not research participants. For example, they can: 1) judge the appropriateness and relevance of the research topic for the host community; 2) assess whether the research methods correctly reflect the standard of care; 3) help ensure that the benefits are shared by the community to which the subjects belong; 4) support activities of the local IRB; and 5) identify and manage non-obvious risks [13–15].

Lack of community involvement could result in higher risks of non-respect for the populations living in the research area, greater potential for overlooking important consequences of the research, and limited uptake of results that are not culturally acceptable. The interruption of trials in some of the sites of the study on tenofovir pre-exposure prophylaxis (PREP) [16] and the poor acceptability of interventions proposed after study completion [17] are just two examples of how things can go wrong when communities are not engaged in research.

Second, community involvement has the potential to increase study efficiency in several steps. The better informed a community is about a study, the easier it will be to recruit subjects. Having a venue where the community can discuss and be regularly updated on the study’s progress helps with study completion, as it decreases the likelihood of interruptions due to community protests. Once the study is completed, such involvement can also improve results uptake, if results are disseminated by community members. All of these are effective approaches to avoid wasting resources and encourage the use of research results.

Third, involving the community in the research is one way to build mutual trust with the population and to show respect to all affected by the research, beyond the study participants. King argues that “listening, acknowledging and being responsive” is an act of respect on the part of the researcher towards the community [15]. In recent experience in Zambia, for instance, the use of CABs has been reported as a way to build not only a link with the community, but also a trusting relationship [18].

In global health research, communities and researchers often are from different cultural backgrounds and countries. They may not only speak different languages, but also have different beliefs and cultural values. This makes it even more difficult for researchers to know and address all the possible implications of their research for the affected populations. Because of this, community participation is an important asset. In many global health settings, there is also the need to extend protection beyond individuals to encompass the community at large [19]. As well, in some settings, researchers must obtain the consent of the community before seeking the consent of individual participants. Furthermore, in global health, the research often pertains to vulnerable populations, such as pregnant women and children, as they are the ones bearing the burden of many diseases of high prevalence in the South.

On the other hand, counterarguments have been raised to illustrate possible adverse consequences of involving communities in research. One is that vulnerable communities could be even more stigmatized when targeted by research [20]. This is certainly true for genetic research [21], where a community could be identified as being at higher risk of some diseases and become stigmatized for it. The research might also discredit some common beliefs held by the community members and with which they strongly identify. In other forms of biomedical research, aside from genetics, the risk might be lower. Still, the disclosure of a study’s results, or even just a researcher’s interest in targeting a community for a specific health issue, could result in its stigmatization by enhancing the idea that this community is more vulnerable or susceptible, or sicker, than others. More commonly, however, in public health and global health research, communities under study are already aware of their health issues and risks and would see the study more as providing an opportunity to get better health care than as posing a risk of stigmatization.

Another argument against community involvement would be that it takes time and resources, which could delay the production of research results, with negative implications for improvement of that same community’s health. At the same time, even the recommendations of well-conducted biomedical research can remain unimplemented if communities that should benefit from the results are not primed to receive and adopt them.

Lastly, it may be argued that not all researchers are necessarily equipped to take on the challenges of establishing community engagement, which may require training and specialized resources. For example, in one successful experience in a malaria vaccine trial [22], a medical anthropologist was involved in conducting the initial assessment, to establish an effective community consultation. However, not all research projects would be able to include this kind of expertise.

While recognizing that any intervention can have unintended consequences, and that realistically not all biomedical researchers would be able to establish long-lasting partnerships with communities, I nevertheless think research could benefit from a paradigm shift in which community involvement is seen as a way to achieve higher research quality.

### Part II: Tools currently available to researchers to promote community involvement and their limitations

Ethics regulatory bodies are increasingly recommending that researchers include a community involvement component in their research, and both public and private funding entities are increasingly requiring community involvement as part of the research plan. In HIV medicine, the US National Institutes of Health (NIH) has supported community involvement in trials both with specific support for community involvement activities from the Division of AIDS and with a specific funding opportunity, the Clinical and Translational Science Award (CTSA).

From a regulatory standpoint, UNAIDS has developed specific guidance for involving communities in HIV vaccine trials [23]. Similarly, there is explicit mention of community involvement in ethical guidance from research organizations such as the HIV Prevention Trials Network (HPTN) [24] and in the H3Africa Guidelines for Community Engagement [25].

Second, there is a push to study the role and efficacy of community involvement in different types of research. Besides the NIH-funded Clinical and Translational Research Program, in the framework of the Grand Challenges in Global Health (GCGH), the Bill & Melinda Gates Foundation (BMGF) [26] dedicated a grant to studying the ethical, social, and cultural issues associated with the research funded by the GCGH initiative [17]. That BMGF program has taken on, among other duties, the study of community involvement in GCGH studies [14]. Similarly, the Wellcome Trust has dedicated funding to examine community involvement in global health research [27]. In the context of global health research, this trend is aligned with a shift towards a more equal power sharing in North–South partnerships and with a larger leadership role being played by researchers in the South.

Third, in the field of HIV research, unique community participation and activism, especially regarding antiretroviral treatment and its accessibility, led to the first institution of community advisory boards (CABs) (1989) and of the Community Constituency Group at the National Institute of Allergy and Infectious Diseases (1993) (NIAID) [28, 29].

Community representation in the trials, with the presence of CABs, has been required by NIH since the late 1980s and early 1990s. The institution and involvement of CABs have since expanded, and they have been used in other areas of medical research, such as malaria research [1, 18, 30].

These initiatives and funding opportunities represent a trend of increasing attention being paid to contributions the community can make to research, as well as its right to be informed. Nevertheless, there is concern that this is not enough of a commitment to build meaningful community links. Authors have noticed that resources for this are limited and are often the first to be sacrificed if there is not enough money for the study [31]. In addition, there is not really a system in place to verify whether community involvement actually occurs in a study (and how), and the literature on evaluations of CABs’ functioning is not yet well established [5, 29, 32], nor is there agreement on how to study their efficiency [28]. Finding measurable universal outcomes that could be considered proxies of efficacy in community involvement is not straightforward, as the ethical objective may be research-specific and vary among the different parties involved in the research. Even when developed, instructions for evaluating community involvement focus more on the process of guaranteeing community involvement and on the level of involvement CABs should have in research. There is no instruction (either uniform or adapted to local contexts) regarding what indicators should be used in addressing ethical issues raised by the use of CABs or regarding how they might best be used to improve post-trial benefit and reduce potential community exploitation [13, 33, 34].

Furthermore, as tools for guaranteeing community involvement in a study, CABs have several limitations.

First, defining what constitutes the “community” is not always straightforward [2, 7, 35]. Definitions differ (Tindana et al. 2007 [14] versus Strauss et al. 2001 [31], for example) and, to some extent, are context specific. In a study involving people from different countries, affected by different conditions, and participating in different types of studies, MacQueen and colleagues [35] concluded that diversity, geographical location, social ties, shared perspectives, and engagement in joint actions were the characteristics that best described a community. As reported by participants of the Community Engagement and Consent Workshop held in Kilifi, Kenya, in 2011, community is often externally defined by the researcher based on the disease and/or the condition(s) studied and the geographical boundaries of the study [36]. As such, the community for a clinical trial on HIV prevention will likely be different from the community for a population-based survey on malaria prevention. If we accept the definition of community as the group of people who share the same risk (and potential benefit) of the research, then the method used in the research (clinical intervention versus observations, for example), as well as the research area (epidemiological research on risk factors, clinical research on treatment, health systems research on strategies, etc.) and the specific topic (malaria, HIV, tuberculosis, poverty and health utilization, diarrheal disease, etc.) would imply different definitions of “community”.

Second, even when there is agreement on the definition of “community” for a given study, the challenge remains of identifying its representatives. Guidelines recommending the institution of CABs do not specify any formal methodology for their composition [28]. Members of CABs can be drawn from either the broad community or a specific population [28, 29]. The first model, a broad community CAB, tends to be less expensive to institute and can be involved in a variety of studies [1, 28]. It may be preferred by researchers as a more affordable option, since it relies on members of the community who already have a leadership role, some of whom also have specific knowledge and are, because of their previous involvement in the community, easier to organize [29]. In some cases, members of this CAB model are elected leaders and therefore already invested by the larger community with the mandate to represent it [1]. In population-specific CABs, on the other hand, members are invited and appointed by the researchers [30]. In this case, the researcher has the difficult task of finding people who can actually represent the community, without perpetuating the marginalization of the most vulnerable members of the community, who may inadvertently be excluded simply because they are not easy to find, nor part of the dominant ethnic or religious group, or for other reasons.

Third, several other limitations to CABs have been raised. These include: lack of clarity regarding who is responsible for identifying and recruiting members who would be considered by the community as suitable representatives and who can work effectively with the researchers; the need for increased awareness in the population regarding the research process, the purpose of the study, and the possibility of having a community voice at the table; the communications challenges (cultural and language barriers are often present between researchers and community members**,** especially in global health, where researchers and communities are from different countries); the social hierarchy and different social statuses within the CAB that can make it difficult for everyone to have a voice; the volunteer nature of community members, with the limitations this can present in terms of time and commitment and in member selection [13]; and the relative independence of CABs from researchers (particularly in cases where researchers are also the main providers of scarce benefits such as drugs, tests, or services, as may be the case especially for marginalized populations or for neglected diseases) [1, 13, 30, 33]. This is particularly true in global health, where the power imbalance between researchers and general population is even more marked. For example, often researchers would be able to provide medical care and devices that are not normally available to the general population with the usual standard of care. This expectation complicates the relationship between community representatives and researchers, as calls for access to better health care besides what is provided to research participants maybe become part of the negotiations [10, 37].

Finally, both scientists and CAB members have raised the issues of insufficient power being given to community representatives and of their actions being largely limited to advising and giving feedback to researchers [1, 13, 30]. A power shift is needed in which CABs can assume a more *intrinsic* role—for instance, participating in setting the study agenda with researchers and evaluating the appropriateness and relative priority of future studies—rather than having a purely *instrumental* role, such as providing guidance in the wording of the informed consent form or helping with recruitment and enrolment [13, 33, 36]. This shift of power would also imply a shift in paradigm, from individual-only protection to community protection [3, 5].

### Part III: Potential solutions

Current limitations in applying community involvement in research could be overcome in different ways.

Some possible solutions are discussed here that could apply to researchers in the field of medicine, mainly with a biomedical background, working on research projects focused on one specific disease, either with interventions or observational research.

On one hand, building on the trend of adopting the CAB model outside HIV medicine, researchers in such areas as malaria, TB, maternal and child health, etc., could be guided and supported in using a similar form of community representation in clinical trials and observational research. On the other hand, a supportive system needs to be in place for researchers to be able to commit time and energy to this issue. Finally, sensitization and specific training for researchers, academics and funders, as well as awareness-raising activities among the general public, could help in shifting the paradigm from protection for participating individuals to protection (and involvement) for the whole affected community [3, 5].

#### Expanding CAB use and tackling current CAB limitations

Despite the limitations discussed above, CABs definitely constitute an effort to include the community at different stages of a clinical trial. Could they be used widely in clinical trials and observational research? Certainly the activism in populations affected by HIV played a strong role in the constitution of CABs in HIV medicine research. Also, one could argue it would be easier (for both communities and researchers) to incorporate community involvement into clinical trials directly linked to the development and use of new drugs than into other kinds of epidemiological studies in which the link to the impact on population health improvement is not always well known or understood by the general public. Moreover, the leading HIV advocacy group, PLWHA (People Living With HIV/AIDS), is a community defined by the disease that affects them, whereas it may be more challenging to elicit the sense of group belonging in the case of other conditions—and even more so when the population under study is not defined in terms of a disease, but of a risk factor. As well, not all sciences may feel that close community involvement is needed, as not all study topics are perceived as sensitive to populations. In particular, observational research is often perceived as potentially less harmful and thus as requiring a lesser level of human subject protection.

In fact, however, even observational research may have harmful consequences. For example, communicating research results to participating individuals can cause distress; healthcare can be withheld if results are *not* communicated to participants, or if action is *not* taken upon them; and, lastly, wide dissemination of results could be stigmatizing for the community. This indicates a clear need for a community safeguard system in observational studies, as well.

If CABs are adopted, then their limitations and the possible perverse consequences of their use [13, 28, 33] need to be addressed. To increase the power of CABs and ensure they play an intrinsic role, for example, Pratt and colleagues suggest three core elements needed to avoid exploitation of the community in which the study takes place [13]. First, procedural requirements must be developed (and respected) for selecting CAB members in ways that ensure appropriate diversity, and the CAB must have an explicit charter giving it the responsibility for preventing exploitation. Second, CAB members need to have sufficient knowledge about how research is conducted, the topic of the study, and the risk of exploitation. Third, CAB members must be given the power to take action if exploitation is recognized, whether by having a direct communication link between the CAB and the ethics committee or by making researchers accountable, in the ethics regulation process, for following CAB advice or justifying why it was not followed [13]. The effectiveness of community involvement is quite complex to evaluate, and assessing it has proven challenging even for groups who have successfully established long-lasting partnerships [37]; research is therefore needed to inform on the most effective methods [33]. Even so, integrating requirements for evaluation into guidelines for CAB use could provide an additional incentive to organize some form of community involvement and to assess whether it makes a difference in the research, with a view towards establishing community involvement in research as the norm.

Making the CABs’ role more central to the research process would require a clear framework and accountability systems, as well as an extended investment in capacity-building in CAB members. It may take a long time, for instance, to develop knowledge on the topic being studied. This is true whether we are establishing a long-term, more open-ended CAB, where members would need continuous education on different health topics, or study-specific CABs, where the challenge would be to train new members for each different study.

In any case, such a commitment to entrust CABs with a more relevant role would require: 1) devoting time and resources to identifying possible members to maximize representation (in the absence of the possibility of election) while reducing power imbalances and avoiding over-representation of some parts of the society; 2) investing time and resources in CAB training and capacity-building, with an honest bi-directional flow of information and learning (i.e., CAB members learning about the disease topic, and researchers learning about societal issues, cultural impacts of the research in that context, etc.); and 3) the willingness to give CABs the mandate to handle agendas and to respond in concrete ways to any cases of injustice raised by the study, and to grant autonomy to the members. This process requires mutual trust and a good level of frequent communication, both of which are slow and labour-intensive to develop. These traits are especially important because CABs need to be dynamic bodies that can adapt to changes in both the community and research needs. For example, whenever there is a new study involving a different vulnerable population, CAB membership will have to be reviewed to ensure appropriate representation of the new group.

Beside CABs, other forms of community representation have also been tried in global health projects. One interesting example has been developed at the Kenya Medical Research Institute (KEMRI) in Kilifi, Kenya, where members drawn from the community-based organizations (CBO) network have served over the years as KEMRI community representatives [10, 37]. This experience illustrates how, in cases of long-standing research infrastructure within a North–South collaboration, it is both possible and beneficial to establish a multifaceted partnership in which community members can participate in different steps of the research projects. Although labour intensive, the partnership nurtured in the KEMRI community representative project has proven quite effective for a research team conducting several studies in the same community [10, 37]. This model is not without limitations; for example, there may be issues in how CBO network members serve as community representatives or relay to the community the information received from and discussed with researchers. Even so, the model in an interesting example of alternatives to CABs for community representation and researcher–community interaction.

For both CABs and other bodies of community representatives, timing is also important in shaping the type of collaboration. If their involvement is sought too late in the process, the community may perceive it as simple consultation with no real commitment to power sharing. If done earlier, it may produce more of a dialogue, which is important to inform research needs and feasibility, but still does not necessarily include a more complex form of partnership, characterized by longer collaboration (established before the study and continued to its completion, results dissemination, and possibly results uptake and implementation). Some of these timings would also require different levels of commitment and resources on the part of researchers, such as the possibility of having access to sufficient funding even before starting the study.

#### Conditions that would need to be in place

Researchers need time, skills, human resources, and funding to be able to build the relationships necessary for meaningful community involvement [10].

However, neither the current funding system nor the academic performance evaluation system seem adequate for these kinds of needs. For example, many authors have suggested that the role of community representatives is more relevant if initiated in the earlier phases of a trial [3, 15, 18]. This means the community’s involvement should start at the beginning of the study, before any decisions have been taken on the protocol and study materials. This would be possible if there were funding to prepare the protocol, in the first place, well before being able to submit a final protocol for funding the study itself. This would require a shift in funding agencies’ rules to make funding available *before* the protocol is submitted.

Furthermore, it would take a supportive academic environment for researchers to be able to devote sufficient time to developing the relationships needed for community involvement. This would mean, for example, redesigning researchers’ performance appraisal systems to take into account collaborations and community involvement as an achievement, rather than merely as a modality used in the study. Accepting such a power shift from the early stages of protocol development all the way to results dissemination could have an impact, for example, on the number and the timing of publications, which are currently an essential part of the evaluation of researchers’ performance in academic institutions. The time and energy the researcher puts into liaising with the community should be considered in career evaluation, counting as a research “result” even if it is only one part of the process leading to the publication of results. Certainly this, as with other methods, would have some limitations, but it could be a way to achieve a more mature community involvement, without leaving it all to researchers’ good intentions.

#### Training, awareness, and evaluation

Community awareness about how research is conducted and the community’s role in it could be raised in many different ways and build on known forms of community consultations (media, open public forums, presentations at public meetings of social or religious groups, etc.) [38]. Training in community involvement rationales, tools, potential barriers, and possible (especially perverse) consequences could become standard for students in all research programs, whether medical research students, epidemiologists, or clinical trialists who are not currently exposed to the existing body of knowledge on CBPR in indigenous populations or who do not work in PAR. It could, for example, become one of the components of good clinical practice for clinical trials, no matter the field studied.

Most importantly, training should be provided early, before researchers have become set in their specialized fields, as this is a common platform of skills needed for different fields of biomedical research, in which researchers are not necessarily equipped in the areas of anthropology, communications, or psychology.

As in any other form of partnership, interpersonal relationships play an important role; even with specific training, not all researchers are suited for or interested in engaging in all the forms of relationship that community participation may require. Different solutions may be appropriate for different people, and some might be a better fit for one specific type of research than for another. For example, a short survey on the community prevalence of a specific non-communicable disease would likely not call for the same community involvement as several longitudinal intervention studies on prevention or treatment of a communicable disease in a vulnerable community in the same population. Far from proposing a comprehensive solution for all researchers and research types, I would argue that, in cases where engaging the population involves complex activities for which the researcher’s training and skills are not suited, working in multidisciplinary teams could be part of the solution, as in the example of the medical anthropologist attached to the malaria trial team [22]. Again, funding and academic environments need to be conducive to this kind of collaboration.

Finally, when training, funding, and recognition are provided for activities aimed at engaging communities, there would need to be in place a system to assess researchers’ practice and effectiveness in working with communities. This would include developing tools to assess the quality of community involvement at different points in the research process. While this evaluation should be kept simple, so as not to overload researchers, academic institutions, and funding bodies with yet more layers of bureaucratic duties, it would underscore the importance of community involvement and could support ongoing reflection and reassessment of the methods and process used.

## Conclusion

In conclusion, agreeing that there is a strong rationale for community involvement in different areas of public health and global health biomedical research is not enough; we also need to put in place conditions that will allow and motivate researchers to actually work towards it, without being penalized in their academic achievements. Expanding the use of a formal organ of community representation, such as the community advisory board, in different fields of epidemiology, observational biomedical research, and clinical trials, as well as increasing researchers’ accountability to these organisms, could be a way of increasing both the voice of the community and research success.

## Abbreviations

BMGF: Bill & Melinda Gates Foundation
CAB: Community advisory board
CBO: Community-based organizations
CBPR: Community based participatory research
CTSA: Clinical and Translational Science Award
GCGH: Grand Challenges in Global Health
HIV: Human immunodeficiency virus
HPTN: HIV prevention trials network
IRB: Institutional review boards (or ethic committees)
KEMRI: Kenya Medical Research Institute in Kilifi, Kenya
NIAID: National Institute of Allergy and Infectious Diseases
NIH: National Institute of Health
PAR: Participatory action research
PLWHA: People living with HIV/AIDS
PREP: Pre-exposure prophylaxis
